# Killer Cell Immunoglobulin-Like Receptor Gene Associations with Autoimmune and Allergic Diseases, Recurrent Spontaneous Abortion, and Neoplasms

**DOI:** 10.3389/fimmu.2013.00008

**Published:** 2013-01-29

**Authors:** Piotr Kuśnierczyk

**Affiliations:** ^1^Laboratory of Immunogenetics and Tissue Immunology, Ludwik Hirszfeld Institute of Immunology and Experimental Therapy, Polish Academy of SciencesWrocław, Poland

**Keywords:** KIR genes, skin disease, rheumatoid arthritis, spontaneous abortion, cancer, viral diseases, viral infections

## Abstract

Killer cell immunoglobulin-like receptors (KIRs) are a family of cell surface inhibitory or activating receptors expressed on natural killer cells and some subpopulations of T lymphocytes. *KIR* genes are clustered in the 19q13.4 region and are characterized by both allelic (high numbers of variants) and haplotypic (different numbers of genes for inhibitory and activating receptors on individual chromosomes) polymorphism. This contributes to diverse susceptibility to diseases and other clinical situations. Associations of *KIR* genes, as well as of genes for their ligands, with selected diseases such as psoriasis vulgaris and atopic dermatitis, rheumatoid arthritis, recurrent spontaneous abortion, and non-small cell lung cancer are discussed in the context of NK and T cell functions.

## Introduction

Killer cell immunoglobulin-like receptors (KIRs) are a family of cell surface receptors. KIR proteins possess two (KIR2D) or three (KIR3D) immunoglobulin-like domains in their extracellular region. KIRs are expressed on natural killer (NK) cells and some subpopulations of T lymphocytes, and therefore may influence the activation of both cell types. They do it either by inhibition of cell activation [inhibitory KIRs, with long (L) cytoplasmic tail – KIR2DL and KIR3DL – which contains immunoreceptor tyrosine-based inhibitory motifs, ITIMs], or by activation of a cell [activating KIRs, KIR2DS, and KIR3DS, with short (S) cytoplasmic tail having no signaling motifs but associated with adapter molecule, DAP12 homodimer, which possesses immunoreceptor tyrosine-based activating motifs, ITAMs]. Upon ligand binding by inhibitory KIR, tyrosine residues in its ITIMs become phosphorylated, which is recognized by a phosphatase, which then dephosphorylates proteins of the signaling pathway, phosphorylated previously due to cell activation. On the other hand, ligand binding by activating KIR results in tyrosine phosphorylation in ITAMs of DAP12 molecule, and this leads to activation of kinases of signaling pathway and cell activation. KIR ligands, where known, are HLA class I molecules (Table 1). All allomorphs of HLA-C are recognized by some inhibitory KIRs, whereas less than 50% of HLA-A and HLA-B allomorphs present in human populations are recognized by KIRs (Parham et al., 2012a). Differing in the amino acid residue in position 80, HLA-C allomorphs fall into two groups, C1 (Asn80) and C2 (Lys80), recognized by KIR2DL2/KIR2DL3 and KIR2DL1/KIR2DS1, respectively (Table 1). As a rule, when inhibitory and activating KIRs have the same or similar HLA specificity (such as KIR2DL1 and KIR2DS1), then binding of inhibitory KIR to its ligand is characterized by higher affinity than binding of activating KIR to the same ligand (Vales-Gomez et al., 1998). This protects normal cells of the body, displaying normal quantity of HLA class I molecules, against the NK cell attack (efficient inhibition), but ensures killing of virus-infected or malignant cells with low or none expression of one or all HLA class I alleles (non-efficient inhibition; “missing-self” theory, Ljunggren and Kärre, 1990).

**Table 1:** **Ligands of KIR molecules (based on Kusnierczyk, 2006; Graef et al., 2009; Campbell and Purdy, 2011; Parham et al., 2012b; and references therein, modified)**.

KIR	Ligand
2DL1	C2
2DL2	C1 and some C2
2DL3	C1
2DL4	HLA-G1
2DL5	Unknown
2DS1	C2
2DS2	Unknown
2DS3	Unknown
2DS4	HLA-A*11, some C1 (*1601 > *0102,1402), C2 (*0502 > 0202 > 0401), and non-identified melanoma antigen
2DS5	Unknown
3DL1	HLA-Bw4
3DL2	HLA-A*03, A*11, and microbial CpG DNA
3DL3	Unknown
3DS1	Unknown (HLA-Bw4?)

HLA-C allele groups (i.e., KIR2D ligands):

C1 (Asn80) – C*01,03,07,08,12,13,1402,1507,1601; HLA-B46.

C2 (Lys80) – C*02,04,05,06,0707,12042,1401,15(without 1507),1602,17.

*KIR* genes are clustered in the leukocyte receptor complex (LRC) of genes located in the 19q13.4 region and are characterized by both allelic (high numbers of variants) and haplotypic (different numbers of genes for inhibitory and activating receptors on individual chromosomes) polymorphism (Parham et al., 2012a). Some *KIR* genes (*KIR3DL2*, *KIR3DL3*, and *KIR2DL4*) are called “framework genes,” because they are present in all haplotypes. Other genes are present only in some of them, in multiple different combinations. Haplotypes consisting mostly of inhibitory genes (so called “A” haplotypes) tend to be associated with lower risk of autoimmune diseases, but higher risk of viral infections than haplotypes (“B” haplotypes) containing several activating *KIR* genes (see Parham, 2005; Khakoo and Carrington, 2006; Kusnierczyk, 2006; Boyton and Altmann, 2007; Campbell and Purdy, 2011)^1^.

Associations of *KIR* and their *HLA* ligand genes have been studied in multiple human diseases, and reviewing all of these here would not be possible. Therefore, selected clinical conditions, most familiar to my laboratory, are summarized and discussed below.

## Skin Diseases: Psoriasis and Atopic Dermatitis

### Psoriasis

Psoriasis is a multifactorial skin disease with autoimmune features, which are manifested by T lymphocyte infiltration to both dermis and epidermis (Lew et al., 2004) and by antipsoriatic activity of immunosuppressants such as recombinant soluble CTLA-4 (Sivamani et al., 2012). Although etiology of this disorder is still not definitely elucidated, it is known that both environmental and genetic factors are involved. Genome-wide association studies revealed at least 13 psoriasis susceptibility loci (*PSORS1-13*)^2^. Among these, the strongest linkage and association was reproducibly described for HLA-Cw*06 allele located on *PSORS1* locus and encoding a ligand for KIR2DL1 and KIR2DS1 receptors (see text footnote 2). Several lines of evidence show contribution of NK or T lymphocytes expressing NK cell receptors, among them KIRs (Gilhar et al., 2002; Liao et al., 2006). Therefore, we examined whether inhibitory or activating *KIR* genes might be associated with susceptibility to psoriasis vulgaris, most common clinical form of this disease. We typed 114 and 116 patients for *HLA-C* alleles and *KIR* genes, respectively, and compared their frequencies with those in 123 unrelated healthy control individuals. We found, first, a strong association of psoriasis with HLA-Cw*06, which was strongest in individuals whose age at disease onset was up to 20 years, and decreased in patient groups with later age at onset (Luszczek et al., 2002). Not surprisingly, we found an association of *KIR2DS1* gene, coding for an activating receptor recognizing HLA-Cw*06 (HLA-Cw*06 belongs to C2 group of HLA-C epitopes), with psoriasis vulgaris. However, in contrast to HLA-Cw*06, association of *KIR2DS1* with psoriasis seemed stronger in higher age at onset values, although the age effect was not significant because of small numbers of patients with late disease onset (Luszczek et al., 2004). Very similar association of *KIR2DS1* (and *KIR2DL5* in addition, which was not analyzed in our study) was simultaneously published for Japanese population, genetically distant from Poles (Suzuki et al., 2004), and confirmed later in Swedish and Brazilian Caucasians with psoriasis vulgaris (Holm et al., 2005; Jobim et al., 2008), but not in Swedes with guttate psoriasis (Holm et al., 2005) or in Taiwanese Chinese with plaque psoriasis (Chang et al., 2006; see Table 2). Interestingly, *KIR2DS1* gene appeared associated also with psoriatic arthritis (Martin et al., 2002b; Holm et al., 2005; Williams et al., 2005; Table 2). In this latter disease, both *KIR2DS1* in the absence of C2, and *KIR2DS2* in the absence of C1 group *HLA-C* alleles were observed associated with psoriatic arthritis (Martin et al., 2002b; Nelson et al., 2004) in addition to other genes (HLA-B*27 and HLA-Cw*0602), although in American population the effect of *KIR2DS1* was independent from C2 presence (Williams et al., 2005).

**Table 2:** **KIR2DS1 gene associations with clinical forms of psoriasis in different ethnic groups**.

Diagnosis	Ethnicity	Number of patients	Number of controls	KIR2DS1 association	Odds ratio^a^	*P*	Reference
PV	Polish	116	123	Yes	5.55	<0.0001	Luszczek et al. (2004)
PV	Swedish	240	372	Yes	1.48	0.0234	Holm et al. (2005)
PV	Braz. Cauc.	79	110	Yes	2.43	0.005	Jobim et al. (2008)
PV	Japanese	96	50	Yes	2.09	<0.05	Suzuki et al. (2004)
PP	Chinese	178	203	No	NA	NS	Chang et al. (2006)
PG	Swedish	80	372	No	NA	NS	Holm et al. (2005)
PA	Swedish	75	372	Yes	1.65	0.0555	Holm et al. (2005)
PA	Canadian	366	299	Yes	1.60	0.004	Martin et al. (2002b)
PA	Am. Cauc.	75	90	Yes	2.41	0.0046	Williams et al. (2005)

*^a^If not given in a publication, then calculated on the basis of its data*.

*Abbreviations: PV, psoriasis vulgaris; PP, plaque psoriasis; PG, guttate psoriasis; PA, psoriatic arthritis; NA, not applicable; NS, non-significant*.

We observed also some effects of other *KIR* genes in psoriatic patients positive for *KIR2DS1*: namely, increased frequency of a deletion variant of *KIR2DS4* and decreased frequency of *KIR2DS3* and *KIR2DS5* gene in comparison to *KIR2DS1*-positive controls (Ploski et al., 2006). A seemingly protective effect of *KIR2DS5* gene presence was seen also in other diseases (Nowak et al., 2010). The deletion variant of *KIR2DS4* gene (Hsu et al., 2002a,b; Maxwell et al., 2002, 2004) potentially encodes a soluble protein, although this has not been proven, and its transcription level is very low (McErlean et al., 2010).

In summary, *KIR2DS1* gene seems to be a major factor in the LRC region contributing to the susceptibility to psoriasis vulgaris and related diseases which are also associated with a gene for its ligand, HLA-Cw*06.

### Atopic dermatitis

Atopic dermatitis (AD) is a chronic or relapsing inflammatory skin disorder of complex etiology, affecting up to 20% of children and often followed later by development of asthma and other allergic diseases. Multiple immunological disturbances were described. Disruption of epidermal barrier increases a susceptibility of AD patients to microbial infections, both bacterial (*Staphylococcus aureus* in most cases) and viral (localized or disseminated infections, most often by *Herpes simplex* virus; De Benedetto et al., 2009; Boguniewicz and Leung, 2011). As NK cells are among many cell types whose activity might be biased in AD, we were interested whether *KIR* and KIR ligand genotype of AD patients would differ from that of healthy persons. We compared *KIR* gene frequencies in a group of 240 patients diagnosed with AD with those in 690 healthy individuals representative for several regions of Poland. Distribution of *KIR* genes in both groups was very similar, with one exception: *KIR2DS1* gene was present less frequently in patients than in controls. This latter observation was confirmed on the second cohort of 201 patients from a different region of Poland (Niepieklo-Miniewska et al., submitted).

The reason why *KIR2DS1* gene has been found associated with psoriasis and psoriatic arthritis (see Psoriasis) but seemingly protective against another inflammatory skin disease, AD, is not clear. Psoriasis is believed to be a Th1-regulated disease, whereas Th2 response dominates in AD (Rabin and Levinson, 2008; von Bubnoff et al., 2010). This division is not so sharp in nature, however (Guttman-Yasky et al., 2011), although microarray analysis confirms it to a great extent (Nomura et al., 2003; Choy et al., 2012). Keratinocytes hyperproliferate in psoriasis but undergo apoptosis in AD (Albanesi et al., 2007; Kastelan et al., 2009; Rebane et al., 2012). Therefore, different KIR2DS1-positive cell subpopulations may contribute to both types of the disease, resulting in opposite associations of *KIR2DS1* gene.

## Rheumatoid Arthritis

Another disease, where *KIR* gene associations were examined, was rheumatoid arthritis (RA). This disorder is a chronic systemic inflammatory polyarthritis affecting about 1% of individuals in Caucasian populations, and T cells contribute to its pathomechanism (Jacob and Jacob, 2012). The involvement of NK cells in RA was also described (Dalbeth et al., 2004; Falgarone et al., 2005). RA is a multifactorial disease, and strongest genetic association was reproducibly shown for so called shared epitope of HLA-DR; however, it explains only one-third to a half of the genetically determined susceptibility to the disease (Bowes and Barton, 2008). The expression of KIR2DS2 molecule on T cells with unusual phenotype, CD4^+^CD28^-^KIR^+^, and an association of *KIR2DS2* but not *KIR2DS1* gene with vascular inflammatory complication of RA were described by Joerg Goronzy’s group (Yen et al., 2001). Other *KIR* genes were not tested by these authors. Therefore, we compared *KIR* gene frequencies in 177 RA patients and 243 control individuals. Although no differences between the whole group of patients and controls were found (Majorczyk et al., 2007) – similarly to simultaneously published study of Northern Irish RA patients (Middleton et al., 2007), nevertheless *KIR2DS2* gene was associated with vasculitis also in our population of patients. In addition, frequency of *KIR2DL2* gene, not tested by Yen et al. (2001), was also increased in vasculitis (Majorczyk et al., 2007). Both these genes are in very strong linkage disequilibrium in Caucasians, which makes differentiation between their effects impossible. However, increased frequency of CD4^+^CD28^−^ T cell clones positive for *KIR2DS2* but negative for *KIR2DL2* gene expression, observed in RA vasculitis by Yen et al. (2001), speaks for the role of the former gene in this complication of RA. Interestingly, CD4^+^CD28^−^ T cells described in RA vasculitis were found only in human cytomegalovirus-seropositive, but not in HCMV-negative RA patients, and their specificity was predominantly directed toward HCMV antigens. If they cross-react weakly with some autoantigens, then KIR2DS2 binding to yet unknown ligands might boost these T cells to autoaggression (van Bergen and Koning, 2010).

Furthermore, the frequencies of *KIR2DS1* and *KIR3DS1* were lower in our patients without bone erosions than in those with erosions and controls. Another interesting finding we made was that RA patients positive for *KIR2DL3* and negative for *KIR2DS3* had earlier disease diagnosis. On the other hand, we observed no associations of *KIR* genes with autoantibodies: rheumatoid factor or anti-cyclic citrullinated peptide (Majorczyk et al., 2007).

Thus, although a susceptibility to RA does not seem to be influenced by particular *KIR* genes, some clinical manifestations of this disease such as vasculitis, bone erosions, and age at onset, are associated with distinct *KIR* genes, which might reflect participation of different subpopulations of T and/or NK cells.

## Immunogenetics of Reproduction

### Recurrent spontaneous abortion

Spontaneous abortion is the most frequent disorder of human pregnancy. Approximately 10–15% of pregnancies end in miscarriage during the first trimester, and even many more spontaneous abortions go undetected. Although most are sporadic and non-recurrent, there is a subset comprising about 1% of all pregnancies which end with recurrent spontaneous abortion (RSA). This is defined as at least three consecutive miscarriages before 20 weeks of gestation (Matthiesen et al., 2012). RSA may have a number of causes (Harris, 2010; Beaman et al., 2012; Matthiesen et al., 2012), as molecular regulation of placentation is very complex, involving both promoting and inhibitory factors secreted by several cell types: decidual stromal cells, decidual macrophages, and decidual NK (dNK) cells (Knoefler and Pollheimer, 2012). Therefore, some of the cases may result from insufficient activity of dNK cells (Harris, 2010; Parham and Guethlein, 2010). NK cells constitute a large leukocyte population in the endometrium and they come in close contact with allogeneic extravillous trophoblast (EVT) cells in early pregnancy decidua, which is necessary for the placentation. EVT cells, in contrast to villous trophoblast cells, do express both maternal and paternal HLA-C molecules (Hiby et al., 2010). Moreover, HLA-C molecules on trophoblast cells form stable heterotrimeric heavy chain/β2-microglobulin/peptide complexes in contrast to decidual and other cells which express not only these heterotrimers, but also free HLA-C heavy chains (Apps et al., 2008). In addition, dNK cells are biased toward expression of HLA-C-binding KIRs (Male et al., 2011). Therefore, their polymorphic KIRs recognizing polymorphic HLA-C molecules inherited from the father by semiallogeneic fetus may play an important role in the outcome of pregnancy. Indeed, recent studies suggest that, in addition to their role in the innate immune response to infection and cancer, KIR-HLA (and particularly KIR-C1/C2) interactions control a proper formation of placenta (Chazara et al., 2011; Colucci et al., 2011; Parham et al., 2012b). Several investigators reported some associations of *KIR* genes with RSA.

First, Varla-Leftherioti et al. (2003) reported a comparison of 26 Greek couples with RSA and 26 fertile couples. They observed twice lower percentage of genotypes containing genes for all three HLA-C binding KIR2DL receptors in RSA couples as compared to control ones, and six times higher fraction of women not possessing KIR2DL genes present in their husbands (Varla-Leftherioti et al., 2003). These findings were confirmed on further 15 spontaneously aborting women compared with 15 women undergoing elective abortion from the Greek population; in addition, in 33.3% of spontaneously aborting women, fetal tissue did not possess a ligand for the inhibitory KIR(s) of the mother (Varla-Leftherioti et al., 2005). However, both these studies were performed on low numbers of patients and controls, and their results were not confirmed by the same authors on larger cohorts involving different ethnic groups (Varla-Leftherioti et al., 2007, 2010). Simultaneously, another group of investigators published a series of thorough studies on the English population, showing that: (a) *KIR* AA genotype frequency was significantly increased, and frequencies of B haplotype-associated *KIR* genes were decreased in 95 RSA cases compared to 269 controls (Hiby et al., 2008); (b) frequency of C2 group *HLA-C* alleles was significantly increased in male partners of RSA women, whereas these women exhibited an increased frequency of *KIR* AA genotype (which contains C2-specific *KIR2DL1* gene; Hiby et al., 2008); and (c) KIR AA frequency was increased in affected mothers (i.e., combined mothers with preeclampsia, RSA, and restricted fetal growth) only when the fetus possessed more C2 genes than the mother, i.e., C2/C2 fetus in C1/C2 mother and C1/C2 fetus in C1/C1 mother (Hiby et al., 2010). This study showed also that the protective effect of haplotype B genes is located in telomeric part of the *KIR* region (TelB, containing *KIR2DS1* gene), whereas no single gene from the haplotype A was found associated with RSA (Hiby et al., 2010). This latter result is not contradicting the association of AA genotype with RSA in England mentioned above, because all of A haplotype genes may appear also in some B haplotypes (Parham, 2005): both a centromeric (CenA) or telomeric (TelA) haplotype A segment may be linked to telomeric (TelB) or centromeric (CenB) segment from a B haplotype, respectively (Cooley et al., 2010; Parham and Guethlein, 2010; Pyo et al., 2010; Chazara et al., 2011), and only CenA/CenA-TelA/TelA genotypes were named “AA” in the past, all other ones being “BB” or “Bx.”

There were also reports on *KIRs* and *HLA-C* in RSA in other populations. Hong et al. (2008) described an association of *KIR2DL2* (a B haplotype gene), but not any other *KIR* gene with RSA in Chinese (Hong et al., 2008). However, this study was made on extremely low number of patients (*N* = 16) and low number of controls (*N* = 41), and the result was not corrected for the number of comparisons. Indeed, another Chinese study on higher numbers of individuals (73 RSA couples and 68 control couples) did not confirm *KIR2DL2* increase, but has rather shown an increase of activating *KIR* genes, *2DS1* and *2DS5* and association of RSA with higher numbers of activating *KIRs* (Wang et al., 2007). Similarly, in 68 Brazilian Caucasian RSA patients, genotypes with five or six activating *KIR* genes were significantly more frequent than in 68 controls (Vargas et al., 2009), although no single *KIR* gene reached significance in frequency distribution in this (Vargas et al., 2009) and other (Witt et al., 2004) study on Brazilian women. In northern India, two *KIR* A haplotype genes, *2DL1* and *2DS4*, were found less frequently in 177 RSA patients than in 200 ethnically matched controls; a combination of *KIR2DL1* in the mother with C2/C2 genotype in both parents was also less frequent in patients, whereas *KIR2DL1* in the mother with C1/C1 genotype in both parents was more frequent in RSA couples than in controls. There were also some combinations of B haplotype-associated genes *KIR2DS1* and *KIR2DS2* with C1 and C2 genotypes which were differently distributed among RSA couples and controls (Faridi and Agrawal, 2011).

We typed 85 Polish Caucasian women with RSA and 117 healthy control women with at least two healthy born children for *KIR* genes and *HLA-C* C1 and C2 markers. We also tested their partners for *HLA-C* alleles and for C1 and C2. Similarly to some other investigators, we did not observe any differences in frequencies of individual *KIR* genes between RSA and control women (Nowak et al., 2011b). However, we found that genotypes with low activating to inhibitory *KIR* ratios were overrepresented in our RSA sample, whereas equilibrium between these two gene kinds seemed to favor a success of pregnancy (Nowak et al., 2009). Nevertheless, AA (most inhibitory) genotype was non-significantly less frequent in RSA than in control (Nowak et al., 2011b), and this result was confirmed by a significant decrease of this genotype in Turkish RSA patients (Ozturk et al., 2012). This result does not, again, seem to contradict the association of low activating to inhibitory *KIR* ratios with RSA mentioned above, because some inhibitory *KIR* genes (*KIR2DL5A*, *KIR2DL5B*) appear only in B haplotypes and therefore contribute to lower activating to inhibitory gene ratio, and some inhibitory *KIR* genes associated with A haplotype appear also in some B haplotypes, decreasing activating to inhibitory *KIR* ratio.

Furthermore, in our study women with AA *KIR* and C1C2 *HLA-C* genotype pregnant with C2C2 males were present in control but completely absent from the RSA group, whereas C1C1 and C2C2 AA women with C2C2 partners were absent from control but present in RSA (Nowak et al., 2011b). Our results are somewhat different from these of Hiby et al. (2010), where C1C2 AA women bearing C2C2 fetus were more frequent in affected pregnancies (RSA included) than in control, whereas we observed C1C2 AA women pregnant with C2C2 males only in control but not in RSA (Nowak et al., 2011b). Also, C1C1 AA women with C1C2 fetus were more frequent in affected group of Hiby et al. (2010), but C1C1 AA mothers with C1C2 father were more frequent in control than in RSA in our sample (Nowak et al., 2011b). These results can not be directly compared, however, as we had no possibility to *HLA-C*-type fetal tissue, particularly in our control, because elective termination of normal pregnancy is legally forbidden in Poland except for some criminal cases and endangered mother’s life. Therefore, in our case, we could predict fetal HLA-C genotype and its parental origin only for some couples (e.g., C1C1 mother and C2C2 father and vice versa).

In summary, results of studies published so far frequently bring conflicting results. The reasons for these discrepancies may be multiple. Some studied populations were genetically very distant, with different *KIR* and *HLA-C* genotype frequencies. This is exemplified by opposite results with *KIR2DS1* association with RSA, negative in England (Hiby et al., 2008, 2010) but positive in China (Wang et al., 2007). In most studies the numbers of patients and controls were low, and extremely low in some reports, as shown in Table 3. Some associations detected in small population samples were not confirmed in larger cohorts. Also, criteria for inclusion of patients and controls were not the same, as we discussed elsewhere (Nowak et al., 2011b). Therefore, there seems to be a need for standardization of studies on genetics of RSA and other pregnancy disorders in different ethnic groups.

**Table 3:** ***KIR* gene associations with recurrent spontaneous abortion**.

Ethnicity	Number of patients	Number of controls	KIR association	Reference
Greeks	26 Couples	26 Couples	2DL1 + 2DL2 + 2DL3 protective	Varla-Leftherioti et al. (2003)
Greeks	15	15	2DL1 + 2DL2 + 2DL3 protective; 2DL – HLA-C mismatch associated	Varla-Leftherioti et al. (2005)
Cauc. + Mongol.	158	81	No significant results	Varla-Leftherioti et al. (2007)
Chinese	73 Couples	68 Couples	2DL1 + C2 in both partners protective; 2DS1 + C2 in both partners protective	Wang et al. (2007)
Argentina	88 Couples	139 Healthy individ.	AA genotype associated; 2DL2 protective	Flores et al. (2007)
Chinese	16	41	2DL2 associated	Hong et al. (2008)
English Caucasian	95 Females; 67 males	269 Females	Female AA associated; male C2 associated	Hiby et al. (2008)
Brasilian Caucasian	68 Couples	68 Couples	Five to six activating KIRs associated	Vargas et al. (2009)
Poles	91	117	Six inhibitory KIRs associated; six activating KIRs protective	Nowak et al. (2009)
English Caucasian	975 RSA + FGR + PE	592	Female AA associated only when fetus has more C2 than mother	Hiby et al. (2010)
Mixed	224	182	No significant results	Varla-Leftherioti et al. (2010)
Asian Indians	177	200	Maternal 2DL1+ both partners C2C2 protective; 2DS2+ both partners C1C1 associated	Faridi and Agrawal (2011)
Poles	85	117	Female AA C1C2+ partner C2C2 strongly protective	Nowak et al. (2011b)
Turks	40	90	Female AA protective	Ozturk et al. (2012)

### An intact *KIR2DL4* gene does not seem necessary for female fertility

*KIR2DL4* differs from other *KIR* genes: (i) it has long cytoplasmic tail which, however, contains only one ITIM sequence; (ii) it has a positively charged arginine residue in the transmembrane region which gives it a possibility to make a complex with the FcεRIγ chain containing an ITAM sequence; (iii) it is not clonally distributed like other KIRs but expressed in all NK cells; (iv) on resting NK cells, it is expressed mostly not at the cell surface but in early endosomes where it can bind a soluble HLA-G molecule (its only known ligand), which is present (in physiological conditions) only on trophoblast cells invading decidua during early pregnancy; and it transmits an activating rather than inhibitory signal inducing cytokine secretion but not cytotoxicity (see a recent review by Rajagopalan and Long, 2012, and references therein).

*KIR2DL4* is one of so called framework genes, present in all *KIR* haplotypes, similarly to *KIR3DL2*, *KIR3DL3*, and *KIR3DP1* (Parham et al., 2012a). Therefore, interaction of KIR2DL4 expressed in dNK cells with HLA-G expressed by trophoblast was suspected to be very important for normal pregnancy (Carosella et al., 2001; Ober et al., 2003; Yan et al., 2007). However, healthy born individuals with defects of *HLA-G* gene were detected (Ober et al., 2003). Nevertheless, they were able to produce truncated form of HLA-G molecules which could substitute for the normal HLA-G (Hunt and Langat, 2009). Although *KIR2DL4* gene was believed to be present in all people worldwide, several individuals lacking this gene were found in different populations in recent years. First one was an African immigrant from Bubi tribe (Bioko island, Equatorial Guinea), a woman who delivered five healthy children and experienced only one spontaneous abortion (Gomez-Lozano et al., 2003). Then, several single cases from Pakistan, Trinidad (also Pakistani by origin), South Turkey, and Solomon Islands were reported to the allele frequency database (Gonzalez-Galarza et al., 2011)^3^. We have also found, in the Polish population of 690 healthy individuals, one woman lacking *KIR2DL4* gene (Nowak et al., 2011a). As her DNA was taken from paternity testing, she must have delivered at least one baby, and therefore was fertile. Interestingly, she had a *KIR* genotype identical to that of the Bubi woman mentioned above: *3DL3-2DS2-2DL2-2DL5B-(del)-2DS5-2DS1-3DL2*. Unfortunately, her personal and family data must have remained anonymous, as required by the Bioethical Committee for samples from paternity testing, therefore neither studies on her family were possible nor data on her further reproductive success were available (Nowak et al., 2011a). However, her case, and particularly that of Bubi individual, indicate that the presence of *KIR2DL4* gene is not absolutely necessary for successful human reproduction, similarly to the presence of intact *HLA-G* gene.

## Neoplastic Diseases

Both NK cell cytotoxic activity (Herberman et al., 1975a,b; Kiessling et al., 1975a,b) and “missing-self” phenomenon (Ljunggren and Kärre, 1990) were discovered in experimental mouse models using tumor cells as targets for NK activity. Transformed neoplastic cells are believed to escape from elimination by cytotoxic T cells due to a reduction or loss of some or all HLA class I (HLA-I) molecules, which in turn exposes them to the attack from NK cells (Ljunggren and Kärre, 1990; Bubenik, 2004; Parham, 2005; Khakoo and Carrington, 2006; Purdy and Campbell, 2009). It has been shown that NK cells may lyze not only cells of established human tumor cell lines but also freshly isolated human tumor cells (Carlsten et al., 2009). Since *KIR* phenotype affects activity of NK cells (and subpopulations of T lymphocytes), one can expect that a prevalence of neoplasms may be influenced by it. Therefore, many investigators examined distribution of *KIR* genes and their ligands as well as their expression in several tumor systems, experimental and clinical (Parham, 2005; Khakoo and Carrington, 2006; van der Meer et al., 2008). We looked whether prevalence of non-small cell lung cancer (NSCLC) might be associated with genes for KIRs and their ligands in the Polish population.

### Non-small cell lung cancer

Non-small cell lung cancer constitutes 85% of lung cancer cases which are a major cause of cancer mortality worldwide. It is a multifactorial disease with a strong environmental (mostly cigarette smoking) influence, but genetic factors also play a role^4^. In NSCLC, NK cells infiltrate rather peritumoral than tumor tissue, in contrast to T lymphocytes (Esendagli et al., 2008; Schneider et al., 2011), and these NK cells which do infiltrate the tumor are predominantly CD56^bright^, negative for NK cell receptors (including KIRs), and exhibit low cytotoxic activity *ex vivo* (Carrega et al., 2008; Esendagli et al., 2008). On the other hand, a tumor-specific cytotoxic T cell clone isolated from tumor infiltrating lymphocytes in an NSCLC patient expressed KIR3DL2 but not other KIRs, and KIR3DL2 had neither stimulating or inhibiting effect on its cytotoxic or interferon-gamma secreting activity (Dorothée et al., 2003). It was also shown that a majority of T cells infiltrating a tumor display T regulatory rather than effector cell phenotype (Esendagli et al., 2008; Schneider et al., 2011). Thus, cells infiltrating malignant areas in NSCLC seem to be poor in KIR expression and cytotoxic activity. Nevertheless, it is conceivable that cytotoxic effector cells, including NK, might eliminate or reduce numbers of potentially metastatic circulating cancer cells, as it has been described for uveal melanoma (Maat et al., 2009).

We typed 269 NSCLC patients for *KIR* and *KIR* ligand genes and compared the results with those of 690 unrelated healthy control individuals, all of them Polish Caucasians. No differences in the distribution of individual *KIR* genes or *AA* and *Bx* genotypes were observed (Wiśniewski et al., 2012). This finding confirms earlier report of Al Omar et al. (2010) in 186 NSCLC cases and 255 controls from England and Northern Ireland. However, we found less frequent prevalence of *HLA-C* *C1/C2* genotype in patients than in controls, whereas both homozygotes were more frequent in patient group (Wiśniewski et al., 2012). This result was discordant with that of Al Omar et al. (2010), who did not observe any association of *HLA-C C1* and *C2* groups (encoding ligands for KIR2DL2/3 and KIR2DL1, respectively) with NSCLC, but found weak association of *HLA-B* *Bw4Thr80* (coding for a ligand for KIR3DL1) which lost significance after correction. This was not, however, visible in our study (Wiśniewski et al., 2012). Interestingly, Al Omar et al. (2010) observed a protective effect of *C1/C2* genotype on the prevalence of NSCLC, but only in the presence of *KIR2DL3* gene, which we have not seen.

The reason for seemingly protective effect of *C1/C2* genotype on NSCLC prevalence needs explanation. HLA-C molecules play two distinct roles in cellular immunity: first, they may present antigenic peptides to CD8^+^ T lymphocytes, although this their function seems less important than that of HLA-A and HLA-B; second, they protect normal cells of the body from the attack of NK cells equipped with HLA-C-binding inhibitory receptors such as KIR2DL1, KIR2DL2, and KIR2DL3. HLA-C molecules participate also in a process called “NK cell education”: these NK cells which possess inhibitory receptors binding self HLA class I molecules, including HLA-C, are “allowed” to mature, whereas NK cell clones devoid of such receptors remain immature and inactive (Björkström et al., 2010; Elliott and Yokoyama, 2011; Schönberg et al., 2011). Therefore, we can imagine that in NSCLC patients, individuals with *C1/C2* genotype may have wider repertoire of antigenic peptides, including tumor antigens, presented to their HLA-C-restricted CD8+ cytotoxic T cells which can eradicate tumor cells. These individuals may also possess wider repertoire of HLA-C-educated NK cell clones which would eliminate these tumor cells which lost HLA-C expression. Transformed cells relatively frequently loose one *HLA-C* allele (Carretero et al., 2008; Mendez et al., 2009), including loss of heterozygosity in lung cancer (So et al., 2005). In *C1/C1* and *C2/C2* homozygotes, this does not change a sensitivity of cancer cells to NK-mediated lysis because they retain the same HLA-C allomorph encoded by the second chromosome and recognized by their mature NK cells. In this respect, *C1/C2* individuals are in a privileged position, because even when a tumor cell retains one *HLA-C* allele, it is still vulnerable to lysis by these NK cells which express KIR recognizing a product of the second allele which had been lost (Maat et al., 2009).

Multivariate analysis revealed an effect of *KIR* and *HLA-C* genotype on the response of our patients to treatment (surgery and/or chemotherapy): *KIR2DL2*+, *KIR2DS2*+, *C1/C1* individuals responded better to therapy and survived longer than patients with other genotypes (median survival time 23 months versus 10 months for patients with other genotypes; Wiśniewski et al., 2012).

Why the effect of KIR2DL2 and KIR2DS2 on treatment response and survival was seen only in the absence of C2 which is not their ligand (or a major ligand in the case of KIR2DL2, see Table 1)? Great majority (about 96%) of our patients (and controls as well) possessed *KIR2DL1* gene whose product strongly interacts with C2^+^ HLA-C molecules (Parham, 2005; see Table 1). Therefore, in C2^+^ patients, NK cells expressing KIR2DL1 would be strongly inhibited and ineffective in C2^+^ tumor eradication. In individuals of *KIR2DL2*^+^, *KIR2DS2*^+^, *C1/C1* genotype this interaction is not possible, therefore NK cells may be activated and kill tumor cells. KIR2DL2 interaction with C1 is much weaker than that of KIR2DL1 with C2 (Parham, 2005), so NK cell activation would not be so strongly inhibited. On the other hand, why *C1/C1* genotype was not protective in the absence of *KIR2DL2* and *KIR2DS2* in our patients, is less clear. Perhaps activating KIR2DS2 receptor, whose ligand is not known (Table 1), is necessary for NK cell-mediated tumor cell lysis in this setting, but NK cells are not sufficiently inhibited by a weak KIR2DL2-C1 interaction. It is also possible that in some circumstances, e.g., when HLA-C C1 molecules are filled with a proper peptide, KIR2DS2 molecule may bind them strongly enough to activate the cell.

Our results suggest that NSCLC patients possessing *KIR2DL2* and *KIR2DS2* genes but not having C2 ligand for KIR2DL1 may respond better to treatment and survive longer than individuals bearing other genotypes. We also indicate that C1/C2 genotype may give some protection from the initiation of this tumor.

## Viral Infections

Viruses infect cells and replicate inside them, forcing cell metabolism to produce abundant amounts of viral proteins. These are degraded to oligopeptides, bound by HLA class I molecules and presented to cytotoxic T cells (CD8^+^) like all other proteins produced within the cell (Neefjes et al., 2011). Therefore, many viruses evolved molecular mechanisms which interfere, in different ways, with cell surface expression of HLA class I molecules (Horst et al., 2011). This makes a virus-producing cell resistant to cytotoxic activity of T cells. On the other hand, the lack of one or all HLA class I molecules makes it susceptible to lysis by NK cells. This, however, depends on the repertoire of KIRs expressed on NK cell clones, which, in turn, depends on *KIR* genotype of the given individual (Parham, 2005; van Bergen and Koning, 2010). Associations of several types of viral infections and resulting human diseases with *KIR* genes have already been studied. Detailed review of the results of these studies would go beyond the acceptable volume of this article, therefore I will only mention here most important findings.

1. HIV-1 infection results in slower progression to AIDS in individuals possessing *KIR3DS1* gene and *HLA-Bw4* variant encoding isoleucine residue in position 80 (Martin et al., 2002a). This discovery stimulated multiple studies which are a topic of recent review (Koerner and Altfeld, 2012).
2. HIV-2 was much less extensively studied, because its prevalence is limited to West Africa, and its infection is more benign than that of HIV-1. No strong correlation with any *KIR* gene (including *KIR3DS1*) was observed, except for a trend for protective effect of *KIR2DL2/KIR2DS2/C1* genotype (Yindom et al., 2010).
3. Both protective and detrimental effects of KIR2DL2 on human T-lymphotropic myelopathy/tropical spastic paraparesis in Japan, depending on HLA allele (Seich al Basatena et al., 2011).
4. Several KIR genes were found to be associated with H1N1 influenza 2009 pandemics (La et al., 2011; Aranda-Romo et al., 2012).
5. Mean number of activating *KIRs* per genotype was lowest in survivors of Ebola virus infection and highest in those with fatal outcome (Wauquier et al., 2010).
6. In human papillomavirus (HPV)-induced cervical intraepithelial neoplasia, protective effect of KIR3DL1 and KIR2DL1 in the presence of their ligands, and increased risk associated with KIR3DS1 was observed in Puerto Ricans and North Americans (Carrington et al., 2005), whereas only protection by KIR2DL5B was found in Swedish sample (Arnheim et al., 2005). In another HPV-induced disease, recurrent respiratory papillomatosis, KIR3DS1 together with KIR2DS1 appeared protective (Bonagura et al., 2010).
7. In HCMV infection of chronic hepatitis (Hepatitis B virus- or hepatitis C virus-induced) an expansion of NKG2C^+^ NK cells selectively expressing KIR2DL2 or KIR2DL3 was described (Beziat et al., 2012). In vasculitis complication of RA, CD4^+^CD28^−^ T cells were observed only in patients infected with HCMV, suggesting a role for HCMV in boosting T cell autoreactivity (van Bergen and Koning, 2010).
8. Hepatitis B virus infection is common worldwide, particularly in China where it is a cause of the highest frequency of hepatocellular carcinoma (HCC) in the world (Zidan et al., 2012). A synergistic effect of a combined genotype *C1C1*^+^/*Bw4-80I*^+^/*KIR2DS4fl*^+^/*KIR2DS4del*^+^ on HCC risk was observed (Pan et al., 2011).
9. The role of *KIR* genes and molecules in HCV infection and HCV-induced HCC was so extensively studied that covering this topic would require a separate article. The protection from low-dose (injection or needle stick) but not from high-dose HCV infection was first described by Khakoo et al. (2004) in British Caucasoids and Afroamericans. The regulation of HCV infection by NK cells was briefly reviewed recently by Brenndörfer and Sällberg (2012).

## General Remarks

The role of polymorphic NK cell receptors, KIRs, recognizing even more polymorphic HLA class I molecules, in human health and disease is gaining a constantly growing interest, and the number of publications is growing exponentially. This review could have touched only a fragment of this field.

## Conflict of Interest Statement

The author declares that the research was conducted in the absence of any commercial or financial relationships that could be construed as a potential conflict of interest.

## Footnotes

^1^http://www.allelefrequencies.net/diseases/dis6001a.asp

^2^http://omim.org/entry/177900

^3^http://www.allelefrequencies.net/

^4^http://omim.org/entry/211980

## References

[B1] Al Omar S. Middleton D. Marshall E. Porter D. Xinarianos G. Raji O. (2010). Associations between genes for killer immunoglobulin-like receptors and their ligands in patients with solid tumors. Hum. Immunol. 71, 976–981 10.1016/j.humimm.2010.06.019 20600442

[B2] Albanesi C. De Pità O. Girolomoni G. (2007). Resident skin cells in psoriasis: a special look at the pathogenetic functions of keratinocytes. Clin. Dermatol. 25, 581–588 10.1016/j.clindermatol.2007.08.013 18021896

[B3] Apps R. Gardner L. Hiby S. E. Sharkey A. M. Moffett A. (2008). Conformation of human leukocyte antigen-C molecules at the surface of human trophoblast cells. Immunology 124, 322–328 10.1111/j.1365-2567.2007.02789.x 18205788 PMC2440826

[B4] Aranda-Romo S. Garcia-Sepulveda C. A. Comas-Garcia A. Lovato-Salas F. Salgado-Bustamante M. Gómez-Gómez A. (2012). Killer-cell immunoglobulin-like receptors (KIR) in severe A (H1N1) 2009 influenza infection. Immunogenetics 64, 653–662 10.1007/s00251-012-0623-3 22652695

[B5] Arnheim L. Dillner J. Sanjeevi C. B. (2005). A population-based cogort study of KIR genes and genotypes in relation to cervical intraepithelial neoplasia. Tissue Antigens 65, 252–259 10.1111/j.1399-0039.2005.00359.x 15730517

[B6] Beaman K. D. Ntrivalas E. Mallers T. M. Jaiswal M. K. Kwask-Kim J. Gilman-Sachs A. (2012). Immune etiology of recurrent pregnancy loss and its diagnosis. Am. J. Reprod. Immunol. 67, 319–325 10.1111/j.1600-0897.2012.01118.x 22380608

[B7] Beziat V. Dalgard O. Asselah T. Halfon P. Bedossa P. Boudifa A. (2012). CMV drives clonal expansion of NKG2C+ NK cells expressing self-specific KIRs in chronic hepatitis patients. Eur. J. Immunol. 42, 447–457 10.1002/eji.201141826 22105371

[B8] Björkström N. K. Riese P. Heuts F. Andersson S. Fauriat C. Ivarsson M. A. (2010). Expression patterns of NKG2A, KIR, and CD57 define a process of CD56dim NK-cell differentiation uncoupled from NK-cell education. Blood 116, 3853–3864 10.1182/blood-2010-04-281675 20696944

[B9] Boguniewicz M. Leung D. Y. M. (2011). Atopic dermatitis: a disease of altered skin barrier and immune dysregulation. Immunol. Rev. 242, 233–246 10.1111/j.1600-065X.2011.01027.x 21682749 PMC3122139

[B10] Bonagura V. R. Du Z. Ashouri E. Luo L. Hatam L. J. DeVoti J. A. (2010). Activating killer cell immunoglobulin-like receptors 3DS1 and 2DS1 protect against developing the severe form of recurrent respiratory papillomatosis. Hum. Immunol. 71, 212–219 10.1016/j.humimm.2009.10.009 19861144 PMC2815039

[B11] Bowes J. Barton A. (2008). Recent advances in the genetics of RA susceptibility. Rheumatology 47, 399–402 10.1093/rheumatology/ken005 18263596

[B12] Boyton R. J. Altmann D. M. (2007). Natural killer cells, killer immunoglobulin-like receptors and human leukocyte antigen class I in disease. Clin. Exp. Immunol. 149, 1–8 10.1111/j.1365-2249.2007.03424.x 17521317 PMC1942026

[B13] Brenndörfer E. D. Sällberg M. (2012). Hepatitis C virus-mediated modulation of cellular immunity. Arch. Immunol. Ther. Exp. (Warsz.) 60, 315–329 10.1007/s00005-012-0184-z 22911132

[B14] Bubenik J. (2004). MHC class I down-regulation: tumor escape from immune surveillance? Int. J. Oncol. 25, 487–491 15254748

[B15] Campbell K. S. Purdy A. K. (2011). Structure/function of human killer cell immunoglobulin-like receptors: lessons from polymorphisms, evolution, crystal structures and mutations. Immunology 132, 315–325 10.1111/j.1365-2567.2010.03398.x 21214544 PMC3044898

[B16] Carlsten M. Malmberg K.-J. Ljunggren H.-G. (2009). Natural killer cell-mediated lysis of freshly isolated human tumor cells. Int. J. Cancer 124, 757–762 10.1002/ijc.24082 19035446

[B17] Carosella E. D. Moreau P. Aractingi S. Rouas-Freiss N. (2001). HLA-G; a shield against inflammatory aggression. Trends Immunol. 22, 553–555 10.1016/S1471-4906(01)02007-5 11574278

[B18] Carrega P. Morandi B. Costa R. Frumento G. Forte G. Altavilla G. (2008). Natural Killer cells infiltrating human non-small cell lung cancer are enriched in CD56 Bright CD16-cells and display an impaired capability to kill tumor cells. Cancer 112, 863–875 10.1002/cncr.23239 18203207

[B19] Carretero R. Romero J. M. Ruiz-Cabello F. Maleno I. Rodriguez F. Camacho F. M. (2008). Analysis of HLA class I expression in progressing and regressing metastatic melanoma lesions after immunotherapy. Immunogenetics 60, 439–447 10.1007/s00251-008-0303-5 18545995

[B20] Carrington M. Wang S. Martin M. P. Gao X. Schiffman M. Cheng J. (2005). Hierarchy of resistance to cervical neoplasia, mediated by combinations of killer immunoglobulin-like receptor and human leukocyte antigen loci. J. Exp. Med. 201, 1069–1075 10.1084/jem.20042158 15809352 PMC2213116

[B21] Chang Y.-T. Chou C.-T. Shiao Y.-M. Lin M.-W. Yu C.-W. Chen C.-C. (2006). The killer cell immunoglobulin-like receptor genes do not confer susceptibility to psoriasis vulgaris independently in Chinese. J. Invest. Dermatol. 126, 2335–2338 10.1038/sj.jid.5700415 16741505

[B22] Chazara O. Xiong S. Moffett A. (2011). Maternal KIR and fetal HLA-C: a fine balance. J. Leukoc. Biol. 90, 703–716 10.1189/jlb.0511227 21873457

[B23] Choy D. F. Hsu D. K. Seshasayee D. Fung M. A. Modrusan Z. Martin F. (2012). Comparative transcriptomic analyses of atopic dermatitis and psoriasis reveal shared neutrophilic inflammation. J. Allergy Clin. Immunol. 130, 1335–1343 10.1016/j.jaci.2012.06.044 22920495 PMC3511596

[B24] Colucci F. Boulenouar S. Kieckbusch J. Moffett A. (2011). How does variability of immune system genes affect placentation? Placenta 32, 539–545 10.1016/j.placenta.2011.05.001 21665273 PMC3202627

[B25] Cooley S. Weisdorf D. J. Guethlein L. A. Klein J. P. Wang T. Le C. T. (2010). Donor selection for natural killer cell receptor genes leads to superior survival after unrelated transplantation for acute myelogenous leukemia. Blood 116, 2411–2419 10.1182/blood-2010-05-283051 20581313 PMC2953880

[B26] Dalbeth N. Gundle R. Davies R. J. O. Lee Y. C. G. McMichael A. J. Callan M. F. C. (2004). CD56bright NK cells are enriched at inflammatory sites and can engage with monocytes in a reciprocal program of activation. J. Immunol. 173, 6418–6426 15528382 10.4049/jimmunol.173.10.6418

[B27] De Benedetto A. Agnihothri R. McGirt L. Y. Bankova L. G. Beck L. A. (2009). Atopic dermatitis: a disease caused by innate immune defects? J. Invest. Dermatol. 129, 14–30 10.1038/jid.2008.259 19078985

[B28] Dorothée G. Echchakir H. Le Maux Chansac B. Vergnon I. El Hage F. Moretta A. (2003). Functional and molecular characterization of a KIR3DL2/p140 expressing tumor-specific T lymphocyte clone infiltrating a human lung carcinoma. Oncogene 22, 7192–7198 10.1038/sj.onc.1206627 14562047

[B29] Elliott J. M. Yokoyama W. M. (2011). Unifying concepts of MHC-dependent natural killer cell education. Trends Immunol. 32, 364–372 10.1016/j.it.2011.06.001 21752715 PMC3151350

[B30] Esendagli G. Bruderek K. Goldmann T. Busche A. Branscheid D. Vollmer E. (2008). Malignant and non-malignant lung tissue areas are differentially populated by natural killer cells and regulatory T cells in non-small cell lung cancer. Lung Cancer 59, 32–40 10.1016/j.lungcan.2007.07.022 17825949

[B31] Falgarone G. Jaen O. Boissier M. C. (2005). Role for innate immunity in rheumatoid arthritis. Joint Bone Spine 72, 17–25 10.1016/j.jbspin.2004.05.013 15681243

[B32] Faridi R. M. Agrawal S. (2011). Killer immunoglobulin-like receptors (KIRs) and HLA-C allorecognition patterns implicative of dominant activation of natural killer cells contribute to recurrent miscarriages. Hum. Reprod. 26, 491–497 10.1093/humrep/deq341 21159685

[B33] Flores A. C. Marcos C. Y. Paladino N. Arruvito L. Williams F. Middleton D. (2007). KIR receptors and HLA-C in the maintenance of pregnancy. Tissue Antigens 69(Suppl. 1), 112–113 10.1111/j.1399-0039.2007.00824.x 17445181

[B34] Gilhar A. Ullmann Y. Kerner H. Assy B. Shalaginov R. Serafimovich S. (2002). Psoriasis is mediated by a cutaneous defect triggered by activated immunocytes: induction of psoriasis by cells with natural killer receptors. J. Invest. Dermatol. 119, 384–391 10.1046/j.1523-1747.2002.01812.x 12190861

[B35] Gomez-Lozano N. de Pablo R. Puente S. Vilches C. (2003). Recognition of HLA-G by the NK cell receptor KIR2DL4 is not essential for human reproduction. Eur. J. Immunol. 33, 639–644 10.1002/eji.200323741 12616484

[B36] Gonzalez-Galarza F. F. Christmas S. Middleton D. Jones A. R. (2011). Allele frequency net: a database and online repository for immune gene frequencies in worldwide populations. Nucleic Acids Res. 39, D913–D919 10.1093/nar/gkq911 21062830 PMC3013710

[B37] Graef T. Moesta A. K. Norman P. J. Abi-Rached L. Vago L. Older-Aguilar A. M. (2009). KIR2DS4 is a product of gene conversion with KIR3DL2 that introduced specificity for HLA-A*11 while diminishing avidity for HLA-C. J. Exp. Med. 206, 2557–2572 10.1084/jem.20091010 19858347 PMC2768862

[B38] Guttman-Yasky E. Nograles K. E. Krueger J. G. (2011). Contrasting pathogenesis of atopic dermatitis and psoriasis – part II: immune cell subsets and therapeutic concepts. J. Allergy Clin. Immunol. 127, 1420–1432 10.1016/j.jaci.2011.01.053 21419481

[B39] Harris L. K. (2010). Review: trophoblast-vascular cell interactions in early pregnancy: how to remodel a vessel. Placenta 24, S93–S98 10.1016/j.placenta.2009.12.012 20060584

[B40] Herberman R. B. Nunn M. E. Holden H. T. Lavrin D. H. (1975a). Natural cytotoxic reactivity of mouse lymphoid cells against syngeneic and allogeneic tumors. II. Characterization of effector cells. Int. J. Cancer 16, 230–239 10.1002/ijc.2910160205 1080480

[B41] Herberman R. B. Nunn M. E. Lavrin D. H. (1975b). Natural cytotoxic reactivity of mouse lymphoid cells against syngeneic and allogeneic tumors. I. Distribution of reactivity and specificity. Int. J. Cancer 16, 216–229 10.1002/ijc.2910160205 50294

[B42] Hiby S. E. Apps R. Sharkey A. M. Farrell L. E. Gardner L. Mulder A. (2010). Maternal activating KIRs protect against human reproductive failure mediated by fetal HLA-C2. J. Clin. Invest. 120, 4102–4110 10.1172/JCI43998 20972337 PMC2964995

[B43] Hiby S. E. Regan L. Lo W. Farrell L. Carrington M. Moffett A. (2008). Association of maternal killer-cell immunoglobulin-like receptors and parental HLA-C genotypes with recurrent miscarriage. Hum. Reprod. 23, 972–976 10.1093/humrep/den011 18263639

[B44] Holm S. J. Sakuraba K. Mallbris L. Wolk K. Ståhle M. Sánchez F. O. (2005). Distinct HLA-C/KIR genotype profile associates with guttate psoriasis. J. Invest. Dermatol. 125, 721–730 10.1111/j.0022-202X.2005.23879.x 16185272

[B45] Hong Y. Wang X. Lu P. Song Y. Lin Q. (2008). Killer immunoglobulinlike receptor repertoire on uterine natural killer cell subsets in women with recurrent spontaneous abortions. Eur. J. Obstet. Gynecol. Reprod. Biol. 140, 218–223 10.1016/j.ejogrb.2008.04.011 18572300

[B46] Horst D. Verweij M. C. Davison A. J. Ressing M. E. Wiertz E. J. (2011). Viral evasion of T cell immunity: ancient mechanisms offering new applications. Curr. Opin. Immunol. 23, 96–103 10.1016/j.coi.2010.11.005 21146386

[B47] Hsu K. C. Chida S. Geraghty D. E. Dupont B. (2002a). The killer cell immunoglobulin-like receptor (KIR) genomic region: gene order, haplotypes and allelic polymorphism. Immunol. Rev. 190, 40–52 10.1034/j.1600-065X.2002.19004.x 12493005

[B48] Hsu K. C. Liu X.-R. Selvakumar A. Mickelson E. O’Reilly R. J. Dupont B. (2002b). Killer Ig-like receptor haplotype analysis by gene content. Evidence for genomic diversity with a minimum of six basic framework haplotypes, each with multiple subsets. J. Immunol. 169, 5123–5134 10.4049/jimmunol.169.9.5118 12391228

[B49] Hunt J. S. Langat D. L. (2009). HLA-G: a human pregnancy-related immunomodulator. Curr. Opin. Pharmacol. 9, 462–469 10.1016/j.coph.2009.05.007 19570712 PMC2747610

[B50] Jacob N. Jacob C. O. (2012). Genetics of rheumatoid arthritis: an impressionist perspective. Rheum. Dis. Clin. North Am. 38, 243–257 10.1016/j.rdc.2012.05.001 22819082

[B51] Jobim M. Jobim L. F. J. Salim P. H. Cestari T. F. Toresan R. Gil B. C. (2008). A study of the killer cell immunoglobulin-like receptor gene KIR2DS1 in a Caucasoid Brazilian population with psoriasis vulgaris. Tissue Antigens 72, 392–396 10.1111/j.1399-0039.2008.01096.x 18643961

[B52] Kastelan M. Prpic-Massari L. Brajac I. (2009). Apoptosis in psoriasis. Acta Dermatovenerol. Croat. 17, 182–186 19818217

[B53] Khakoo S. I. Carrington M. (2006). KIR and disease: a model system or system of models? Immunol. Rev. 214, 186–201 10.1111/j.1600-065X.2006.00459.x 17100885

[B54] Khakoo S. Thio C. L. Martin M. P. Brooks C. R. Gao X. Astemborski J. (2004). HLA and NK cell inhibitory receptor genes in resolving hepatitis C virus infection. Science 305, 872–874 10.1126/science.1097670 15297676

[B55] Kiessling R. Klein E. Wigzell H. (1975a). “Natural” killer cells in the mouse. II. Cytotoxic cells with specificity for mouse Moloney leukemia cells. Characteristics of the killer cell. Eur. J. Immunol. 5, 117–121 10.1002/eji.1830050208 1086218

[B56] Kiessling R. Klein E. Wigzell H. (1975b). “Natural” killer cells in the mouse. I. Cytotoxic cells with specificity for mouse Moloney leukemia cells. Specificity and distribution according to genotype. Eur. J. Immunol. 5, 112–117 10.1002/eji.1830050208 1234049

[B57] Knoefler M. Pollheimer J. (2012). IFPA award in placentology lecture: molecular regulation of human trophoblast invasion. Placenta 33, S55–S62 10.1016/j.placenta.2011.09.019 22019198 PMC3272142

[B58] Koerner C. Altfeld M. (2012). Role of KIR3DS1 in human diseases. Front. Immunol. 3:326 10.3389/fimmu.2012.00326 23125843 PMC3485674

[B59] Kusnierczyk P. (2006). “Sensing the self: structure, genetics, biological function and possible disease associations of KIR genes and molecules,” in Leading-Edge Immunology Research, ed. Veskler B. A. (New York: Nova Science Publishers, Inc.), 95–125

[B60] La D. Czarnecki C. El-Gabalawy H. Kumar A. Meyers A. F. A. Bastien N. (2011). Enrichment of variations in KIR3DL1/S1 and KIR2DL2/L3 among H1N1/09 ICU patients: an exploratory study. PLoS ONE 6:e29200 10.1371/journal.pone.0029200 22216211 PMC3247251

[B61] Lew W. Bowcock A. M. Krueger J. G. (2004). Psoriasis vulgaris: cutaneous lymphoid tissue supports T-cell activation and ‘Type 1’ inflammatory gene expression. Trends Immunol. 25, 295–305 10.1016/j.it.2004.03.006 15145319

[B62] Liao Y. H. Jee S. H. Sheu B. C. Huang Y. L. Tseng M. P. Hsu S. M. (2006). Increased expression of the natural killer cell inhibitory receptor CD94/NKG2A and CD158b on circulating and lesional T cells in patients with chronic plaque psoriasis. Br. J. Dermatol. 155, 318–324 10.1111/j.1365-2133.2006.07301.x 16882169

[B63] Ljunggren H.-G. Kärre K. (1990). In search of the ‘missing self’: MHC molecules and NK cell recognition. Immunol. Today 11, 237–244 10.1016/0167-5699(90)90097-S 2201309

[B64] Luszczek W. Kubicka W. Cislo M. Nockowski P. Manczak M. Woszczek G. (2002). Strong association of HLA-Cw6 allele with juvenile psoriasis in Polish patients. Immunol. Lett. 85, 59–64 10.1016/S0165-2478(02)00212-2 12505198

[B65] Luszczek W. Manczak M. Cislo M. Nockowski P. Wisniewski A. Jasek M. (2004). Gene for the activating natural killer cell receptor, KIR2DS1, is associated with susceptibility to psoriasis vulgaris. Hum. Immunol. 65, 758–766 10.1016/j.humimm.2004.05.008 15310528

[B66] Maat W. van der Slik A. R. Verhoeven D. H. J. Alizadeh B. Z. Ly L. V. Verduijn W. (2009). Evidence for natural killer cell-mediated protection from metastasis formation in uveal melanoma patients. Invest. Ophthalmol. Vis. Sci. 50, 2888–2895 10.1167/iovs.08-2733 19234348

[B67] Majorczyk E. Pawlik A. Luszczek W. Nowak I. Wisniewski A. Jasek M. (2007). Associations of killer cell immunoglobulin-like receptor genes with complications of rheumatoid arthritis. Genes Immun. 8, 678–683 10.1038/sj.gene.6364433 17882223

[B68] Male V. Sharkey A. Masters L. Kennedy P. R. Farrell L. E. Moffett A. (2011). The effect of pregnancy on the uterine NK cell KIR repertoire. Eur. J. Immunol. 41, 3017–3027 10.1002/eji.201141445 21739430 PMC3262970

[B69] Martin M. P. Gao X. Lee J. H. Nelson G. W. Detels R. Goedert J. J. (2002a). Epistatic interaction between KIR3DS1 and HLA-B delays the progression to AIDS. Nat. Genet. 31, 429–434 12134147 10.1038/ng934

[B70] Martin M. Nelson G. Lee J.-H. Pellett F. Gao X. Wade J. (2002b). Cutting edge: susceptibility to psoriatic arthritis: influence of activating killer Ig-like receptor genes in the absence of specific HLA-C alleles. J. Immunol. 169, 2818–2822 12218090 10.4049/jimmunol.169.6.2818

[B71] Middleton D. Meenagh A. Wright G. D. (2007). No association in frequency of KIR receptors in patients with rheumatoid arthritis from Northern Ireland. Tissue Antigens 69, 577–582 10.1111/j.1399-0039.2006.00792.x 17498267

[B72] Matthiesen L. Kalkunte S. Sharma S. (2012). Multiple pregnancy failures: an immunological paradigm. Am. J. Reprod. Immunol. 67, 334–340 10.1111/j.1600-0897.2012.01121.x 22380628

[B73] Maxwell L. D. Wallace A. Middleton D. Curran M. D. (2002). A common KIR2DS4 deletion variant in the human that predicts a soluble KIR molecule analogous to the KIR1D molecule observed in the rhesus monkey. Tissue Antigens 60, 254–258 10.1034/j.1399-0039.2002.600307.x 12445308

[B74] Maxwell L. D. Williams F. Gilmore P. Meenagh A. Middleton D. (2004). Investigation of killer cell immunoglobulin-like receptor gene diversity: II. KIR2DS4. Hum. Immunol. 65, 613–621 10.1016/j.humimm.2004.02.028 15219381

[B75] McErlean C. Gonzalez A. A. Cunningham R. Meenagh A. Shovlin T. Middleton D. (2010). Differential RNA expression of KIR alleles. Immunogenetics 62, 431–440 10.1007/s00251-010-0449-9 20454893

[B76] Mendez R. Aptsiauri N. Del Campo A. Maleno I. Cabrera T. Ruiz-Cabello F. (2009). HLA and melanoma: multiple alterations in HLA class I and II expression in human melanoma cell lines from ESTDAB cell bank. Cancer Immunol. Immunother. 58, 1507–1515 10.1007/s00262-009-0701-z 19340423 PMC11030131

[B77] Neefjes J. Jongsma M. L. Paul P. Bakke O. (2011). Towards a systems understanding of MHC class I and MHC class II antigen presentation. Nat. Rev. Immunol. 11, 823–836 22076556 10.1038/nri3084

[B78] Nelson G. W. Martin M. P. Gladman D. Wade J. Trowsdale J. Carrington M. (2004). Cutting edge: heterozygote advantage in autoimmune disease: hierarchy of protection/susceptibility conferred by HLA and killer Ig-like receptor combinations in psoriatic arthritis. J. Immunol. 173, 4273–4276 15383555 10.4049/jimmunol.173.7.4273

[B79] Nomura I. Gao B. Boguniewicz M. Darst M. A. Travers J. B. Leung D. Y. M. (2003). Distinct patterns of gene expression in the skin lesions of atopic dermatitis and psoriasis: a gene microarray analysis. J. Allergy Clin. Immunol. 112, 1195–1202 10.1016/j.jaci.2003.08.049 14657882

[B80] Nowak I. Majorczyk E. Ploski R. Senitzer D. Sun J. Y. Kusnierczyk P. (2011a). Lack of KIR2DL4 gene in a fertile Caucasian woman. Tissue Antigens 78, 115–119 10.1111/j.1399-0039.2011.01711.x 21623736

[B81] Nowak I. Malinowski A. Tchórzewski H. Barcz E. Wilczynski J. R. Banasik M. (2011b). HLA-C C1C2 heterozygosity may protect women bearing the killer immunoglobulin-like receptor AA genotype from spontaneous abortion. J. Reprod. Immunol. 88, 32–37 10.1016/j.jri.2010.11.001 21134695

[B82] Nowak I. Majorczyk E. Wisniewski A. Pawlik A. Magott-Procelewska M. Passowicz-Muszynska E. (2010). Does the KIR2DS5 gene protect from some human diseases? PLoS ONE 5:e12381 10.1371/journal.pone.0012381 20865034 PMC2928722

[B83] Nowak I. Malinowski A. Tchórzewski H. Barcz E. Wilczynski J. R. Grybos M. (2009). Frequencies of killer immunoglobulin-like receptor genotypes influence susceptibility to spontaneous abortion. J. Appl. Genet. 50, 391–398 10.1007/BF03195699 19875891

[B84] Ober C. Aldrich C. L. Chervenova I. Billstrand C. Rahimov F. Gray H. L. (2003). Variation in the HLA-G promoter region influences miscarriage rates. Am. J. Hum. Genet. 72, 1425–1435 10.1086/375501 12721954 PMC1180303

[B85] Ozturk O. G. Sahin G. Ziyanoglu Karacor E. D. Kucukgoz U. (2012). Evaluation of KIR genes in recurrent miscarriage. J. Assist. Reprod. Genet. 29, 933–938 10.1007/s10815-012-9811-1 22669573 PMC3463666

[B86] Pan N. Jiang W. Sun H. Miao F. Qiu J. Jin H. (2011). KIR and HLA loci are associated with hepatocellular carcinoma development in patients with hepatitis B virus infection: a case-control study. PLoS ONE 6:e25682 10.1371/journal.pone.0025682 21998681 PMC3187788

[B87] Parham P. (2005). MHC class I molecules and KIRs in human history, health and survival. Nat. Rev. Immunol. 5, 201–214 10.1038/nri1570 15719024

[B88] Parham P. Guethlein L. A. (2010). Pregnancy immunogenetics: NK cell education in the womb? J. Clin. Invest. 120, 3801–3804 10.1172/JCI44559 20972330 PMC2964998

[B89] Parham P. Norman P. J. Abi-Rached L. Guethlein L. A. (2012a). Human-specific evolution of killer cell immunoglobulin-like receptor recognition of major histocompatibility complex class I molecules. Philos. Trans. R. Soc. Lond. B Biol. Sci. 367, 800–811 10.1098/rstb.2011.0266 22312047 PMC3267113

[B90] Parham P. Norman P. J. Abi-Rached L. Hilton H. G. Guethlein L. A. (2012b). Review: immunogenetics of human placentation. Placenta 33, S71–S80 10.1016/j.placenta.2011.11.020 22177321 PMC3677947

[B91] Ploski R. Luszczek W. Kusnierczyk P. Nockowski P. Cislo M. Krajewski P. (2006). A role for KIR gene variants other than KIR2DS1 in confering susceptibility to psoriasis. Hum. Immunol. 67, 521–526 10.1016/j.humimm.2006.04.001 16829306

[B92] Purdy A. K. Campbell K. S. (2009). Natural killer cells and cancer: regulation by the killer cell Ig-like receptors (KIR). Cancer Biol. Ther. 8, 13–22 10.1158/1535-7163.TARG-09-C13 19923897 PMC2825280

[B93] Pyo C.-W. Guethlein L. A. Vu Q. Wang R. Abi-Rached L. Norman P. J. (2010). Different patterns of evolution in the centromeric and telomeric regions of group A and group B haplotypes of the human killer cell Ig-like receptor locus. PLoS ONE 5:e15115 10.1371/journal.pone.0015115 21206914 PMC3012066

[B94] Rabin R. L. Levinson A. I. (2008). The nexus between atopic disease and autoimmunity: a review of the epidemiological and mechanistic literature. Clin. Exp. Immunol. 153, 19–30 10.1111/j.1365-2249.2008.03679.x 18505431 PMC2432093

[B95] Rajagopalan S. Long E. O. (2012). KIR2DL4 (CD158d): an activation receptor for HLA-G. Front. Immun. 3:258 10.3389/fimmu.2012.00258 PMC3422731 22934097

[B96] Rebane A. Zimmermann M. Aab A. Baurecht H. Koreck A. Karelson M. (2012). Mechanisms of IFN-γ-induced apoptosis of human skin keratinocytes in patients with atopic dermatitis. J. Allergy Clin. Immunol. 129, 1297–1306 10.1016/j.jaci.2012.02.020 22445417

[B97] Schneider T. Kimpfler S. Warth A. Schnabel P. A. Dienemann H. Schadendorf D. (2011). Foxp3+ regulatory T cells and natural killer cells distinctly infiltrate primary tumors and draining lymph nodes in pulmonary adenocarcinoma. J. Thorac. Oncol. 6, 432–438 10.1097/JTO.0b013e31821c421d 21258248

[B98] Schönberg K. Sribar M. Enczmann J. Fischer J. C. Uhrberg M. (2011). Analyses of HLA-C-specific KIR repertoires in donors with group A and B haplotypes suggest a ligand-instructed model of NK cell receptor acquisition. Blood 117, 98–107 10.1182/blood-2010-03-273656 20935255

[B99] Seich al Basatena N.-K. MacNamara A. Vine A. M. Thio C. L. Astemborski J. Usuku K. (2011). KIR2DL2 enhances protective and detrimental HLA class I-mediated immunity in chronic viral infection. PLoS Pathog. 7:e1002270 10.1371/journal.ppat.1002270 22022261 PMC3192839

[B100] Sivamani R. K. Goodarzi H. Shirakawa Garcia M. Raychaudhuri S. P. Wehrli L. N. Ono Y. (2012). Biologic therapies in the treatment of psoriasis: a comprehensive evidence-based basic science and clinical review and a practical guide to tuberculosis monitoring. Clin. Rev. Allergy Immunol. 10.1007/s12016-012-8301-8307 22311162

[B101] So T. Takenoyama M. Mizukami M. Ichiki Y. Sugaya M. Hanagiri T. (2005). Haplotype loss of HLA class I antigen as an escape mechanism from immune attack in lung cancer. Cancer Res. 65, 5945–5952 10.1158/0008-5472.CAN-04-3787 15994973

[B102] Suzuki Y. Hamamoto Y. Ogasawara Y. Ishikawa K. Yoshikawa Y. Sasazuki T. (2004). Genetic polymorphisms of killer cell immunoglobulin-like receptors are associated with susceptibility to psoriasis vulgaris. J. Invest. Dermatol. 122, 1133–1136 10.1111/j.0022-202X.2004.22517.x 15140215

[B103] Vales-Gomez M. Reyburn H. T. Erskine R. A. Strominger J. (1998). Differential binding to HLA-C of p50-activating and p58-inhibitory natural killer cell receptors. Proc. Natl. Acad. Sci. U.S.A. 95, 14326–14331 10.1073/pnas.95.24.14326 9826699 PMC24372

[B104] van Bergen J. Koning F. (2010). The tortoise and the hare: slowly evolving T-cell responses take hastily evolving KIR. Immunology 131, 301–309 10.1111/j.1365-2567.2010.03337.x 20722764 PMC2996550

[B105] van der Meer A. Schaap N. P. M. Schattenberg A. V. M. B. van Cranenbroek B. Tijssen H. J. Joosten I. (2008). KIR2DS5 is associated with leukemia free survival after HLA identical stem cell transplantation in chronic myeloid leukemia patients. Mol. Immunol. 45, 3631–3638 10.1016/j.molimm.2008.04.016 18555529

[B106] Vargas R. G. Bompeixe E. P. Pinhode Franca P. Marques de Moraes M. da Graca Bicalho M. (2009). Activating killer cell immunoglobulin-like receptor genes’ association with recurrent miscarriage. Am. J. Reprod. Immunol. 62, 34–43 10.1111/j.1600-0897.2009.00709.x 19527230

[B107] Varla-Leftherioti M. Keramitsoglou T. Spyropoulou-Vlachou M. Papadimitropoulos M. Kontopoulou-Antonopoulou V. Tsekoura C. (2007). 14th International HLA and Immunogenetics Workshop: report from the reproductive immunology component. Tissue Antigens 69(Suppl. 1), 297–303 10.1111/j.1399-0039.2006.00782.x 17445221

[B108] Varla-Leftherioti M. Keramitsoglu T. Parapanissiou E. Kurpisz M. Kontopoulou-Antonopoulou V. Tsekoura C. (2010). HLA-DQA1*0505 sharing and Killer immunoglobulin-like receptors in sub fertile couples: report of the 15th International Histocompatibility Workshop. Tissue Antigens 75, 668–672 10.1111/j.1399-0039.2010.01451.x 20210919

[B109] Varla-Leftherioti M. Spyropoulou-Vlachou M. Keramitsoglou T. Papadimitropoulos M. Tsekoura C. Graphou O. (2005). Lack of the appropriate natural killer cell inhibitory receptors in women with spontaneous abortion. Hum. Immunol. 66, 65–71 10.1016/j.humimm.2004.10.005 15620464

[B110] Varla-Leftherioti M. Spyropoulou-Vlachou M. Niokou D. Keramitsoglou T. Darlamitsou A. Tsekoura C. (2003). Natural killer (NK) cell receptors’ repertoire in couples with recurrent spontaneous abortions. Am. J. Reprod. Immunol. 49, 183–191 10.1034/j.1600-0897.2003.00018.x 12797525

[B111] von Bubnoff D. Andrès E. Hentges F. Bieber T. Michel T. Zimmer J. (2010). Natural killer cells in atopic and autoimmune diseases of the skin. J. Allergy Clin. Immunol. 125, 60–68 10.1016/j.jaci.2009.11.020 20109737

[B112] Wang S. Zhao Y.-R. Jiao Y.-L. Wang L.-C. Li J.-F. Cui B. (2007). Increased activating killer immunoglobulin-like receptor genes and decreased specific HLA-C alleles in couples with recurrent spontaneous abortion. Biochem. Biophys. Res. Commun. 360, 696–701 10.1016/j.bbrc.2007.06.097 17617375

[B113] Wauquier N. Padilla C. Becquart P. Leroy E. Wieillard V. (2010). Association of KIR2DS1 and KIR2DS3 with fatal outcome in Ebola virus infection. Immunogenetics 62, 767–771 10.1007/s00251-010-0480-x 20878400 PMC2978320

[B114] Williams F. Meenagh A. Sleator C. Cook D. Fernandez-Vina M. Bowcock A. M. (2005). Activating killer cell immunoglobulin-like receptor gene KIR2DS1 is associated with psoriatic arthritis. Hum. Immunol. 66, 836–841 10.1016/j.humimm.2005.04.005 16112031

[B115] Wiśniewski A. Jankowska R. Passowicz-Muszyńska E. Wiśniewska E. Majorczyk E. Nowak I. (2012). KIR2DL2/S2 and HLA-C C1C1 genotype is associated with better response to treatment and prolonged survival of patients with non-small cel lung cancer in a Polish Caucasian population. Hum. Immunol. 73, 927–931 10.1016/j.humimm.2012.07.323 22836042

[B116] Witt C. S. Goodridge J. Gerbase-Delima M. G. Daher S. Christiansen F. T. (2004). Maternal KIR repertoire is not associated with recurrent spontaneous abortion. Hum. Reprod. 19, 2653–2657 10.1093/humrep/deh483 15333596

[B117] Yan W.-H. Lin A. Chen B.-G. Zhou M.-Y. Dai M.-Z. Chen X.-J. (2007). Possible roles of KIR2DL4 expression on uNK cells in human pregnancy. Am. J. Reprod. Immunol. 57, 233–242 10.1111/j.1600-0897.2007.00469.x 17362384

[B118] Yen J. H. Moore B. E. Nakajima T. Scholl D. Schaid D. J. Weyand C. M. (2001). Major histocompatibility complex class I-recognizing receptors are disease risk genes in rheumatoid arthritis. J. Exp. Med. 193, 1159–1167 10.1084/jem.193.10.1159 11369787 PMC2193323

[B119] Yindom L.-M. Leligdowicz A. Martin M. P. Gao X. Qi Y. Zaman S. M. A. (2010). Influence of HLA class I and HLA-KIR compound genotypes on HIV-2 infection and markers of disease progression in a Manjako community in West Africa. J. Virol. 84, 8202–8208 10.1128/JVI.00116-10 20519398 PMC2916551

[B120] Zidan A. Scheuerlein H. Schuele S. Settmacher U. Rauchfuss F. (2012). Epidemiological pattern of hepatitis B and hepatitis C as etiological agents for hepatocellular carcinoma in Iran and worldwide. Hepat. Mon. 12, e6894 23233864 10.5812/hepatmon.6894 PMC3517809

